# Mimicking Electromagnetic Wave Coupling in Tokamak Plasma with Fishnet Metamaterials

**DOI:** 10.1038/s41598-018-24250-0

**Published:** 2018-04-11

**Authors:** K. Rustomji, R. Abdeddaim, J. Achard, M. Chmiaa, E. Georget, M. Goniche, W. Helou, J. Hillairet, S. Enoch, G. Tayeb

**Affiliations:** 1 Aix Marseille Univ, CNRS, Centrale Marseille, Institut Fresnel, Marseille, France; 2 Centre for Ultrahigh Bandwidth Devices for Optical Systems and Institute of Photonics and Optical Science, School of Physics, University of Sydney, Sydney, NSW 2006 Australia; 3 CEA, IRFM, F-13108 St-Paul-Lez-Durance, France; 4 CEA-Saclay, DRF/I2BM/Neurospin/UNIRS, 91191 Gif-sur-Yvette Cedex, France

## Abstract

This paper reports a fishnet hyperbolic metamaterial that mimics the electromagnetic properties of magnetically confined plasma. These electromagnetic properties are strongly anisotropic and different from any conventional material, therefore cannot be mimicked by bulk materials. The structure is made of a stack of thin copper grids spaced by Rohacell foam. We numerically and experimentally show that this kind of structuration matches well the properties of a homogeneous plasma. This solution breaks a long-lasting bottleneck and will accelerate the development of high-frequency heating systems to be used in nuclear fusion.

## Introduction

Tokamaks are expected to become the standard choice for magnetic confinement devices to produce nuclear fusion, an environmentally friendly solution for future large-scale energy supply. International projects such as ITER are leading the way in this field. To transfer energy into the plasma, dedicated radio frequency antennas are required. Their development and optimization require the availability of a loading material that mimics the plasma. Until now, there exists no realistic load able to simulate a tokamak plasma, and consequently no way to test the antennas that will be used to create the plasma.

The problem is to transfer power from the antenna to the plasma, and test these antennas under relevant conditions. The power transfer is mainly related to the characteristics of the plasma at the tokamak edge (location of the antenna’s mouth).

In this paper, the idea is to design and build a metamaterial to mimic the plasma. It appears legitimate to simplify the characteristics of the edge plasma by tending towards hyperbolic materials. Our design allowed us to build a metamaterial that overcomes the problem of spatial dispersion encountered in previous studies. We carried out numerical simulations and measurements, which confirm the validity of this concept. As a result, we now have a way to characterize and test the antennas. One of the other advantages is that these tests can be done at low power, without the need for elaborate sources or cooling. It should be noted that we do not intend to model with our metamaterials actual tokamak plasma, but only simplified plasma from the tokamak edge that retains the essential properties related to the coupling of the waves launched by the antenna to the plasma.

## Design and Fabrication

The coupling of Lower Hybrid Range of Frequencies (LHRF, 1–8 GHz) waves to strongly magnetized plasmas is a critical issue for tokamaks as it often limits the RF power, which can be transferred from the antenna to the plasma. Development of new types of antennas to improve the ability of the antenna to handle large power in stationary conditions, as required on a fusion reactor, is hampered by the long and costly delay between the design and the feedback from experiments on large facilities such as tokamaks. Numerical codes that model both the plasma and the antenna are now available and provide an accurate characterization of the RF coupling. Before a full-scale test of the antenna on the plasma, the test of a mock-up with a load mimicking the plasma is believed to be a step forward by reducing the risks and accelerating the development process.

**Figure 1:**
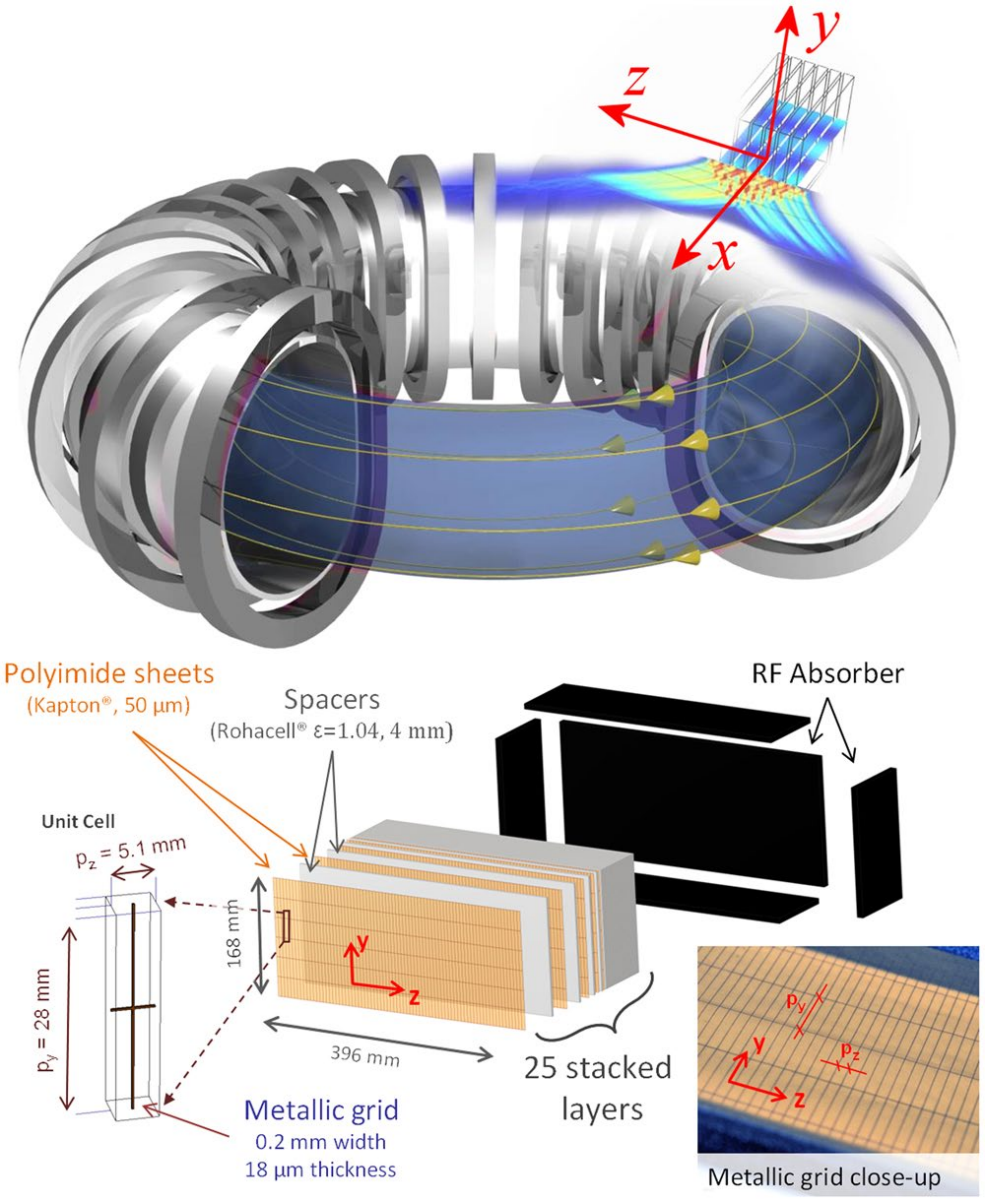
Top: sketch of an antenna made of 6 waveguides, heating tokamak plasma (not to scale). The yellow lines depict the plasma current. For a picture of an actual set of waveguides feeding a tokamak, see the supplementary information. Bottom: schematic view of the metamaterial fishnet load.

The magnetic configuration of a tokamak requires a strong DC current flowing along the plasma ring^1^. Generally, this current is inductively driven by the solenoid located on the tokamak axis. However, in order to achieve continuous operation of the reactor, an external source of current is needed. RF waves emitted from the plasma periphery are well fitted for this task when the wavevector spectrum is properly chosen in order to transfer the wave energy to the electrons of the plasma current (Fig. 1). Among these waves, LHRF waves are a good candidate, thanks to their high current drive efficiency^2^.

The dielectric tensor of magnetized plasma in the cold plasma approximation can be written in the form:^3^

$${\boldsymbol{\varepsilon }}={{\rm{\varepsilon }}}_{0}\,[\begin{array}{ccc}{{\rm{\varepsilon }}}_{\perp } & {{\rm{\varepsilon }}}_{{\rm{A}}} & 0\\ {{\rm{\varepsilon }}}_{{\rm{A}}} & {{\rm{\varepsilon }}}_{\perp } & 0\\ 0 & 0 & {{\rm{\varepsilon }}}_{//}\end{array}]$$

where the subscript//denotes the direction of the confinement magnetic field, i.e., the z-axis on Fig. 1.

In the case of LHRF waves of frequency ω_0_/2π in the 1–8 GHz range, the electron density in the vicinity of the antenna has to be optimized to minimize the RF power reflected towards the generator and the electric field in the waveguides. This is achieved when the density *n*_e_ exceeds the cut-off density (*n*_cut-off_ = (ε_0_ *m*_e_/*e*^2^) ω_0_^2^) typically by a factor of 3. Under such conditions, the non-diagonal term ε_A_ is small (ε_A_ ~ 0.1) and ε_⊥_ differs from 1 by less than 1%. Consequently, **ε** can be simplified using ε_⊥_ ≈ 1 and ε_A_ ≈ 0. The third parameter ε_//_ is approximatively reduced to a Drude model ε_//_ = 1 − ω_pe_^2^/ω_0_^2^ = 1 − *n*_e_/*n*_cut-off_ where ω_pe_/2π is the plasma frequency (ω_pe_^2^ = *n*_e_ *e*^2^/(ε_0_ *m*_e_)).

Good coupling conditions of the wave to the plasma are achieved when the relative permittivity in the direction of the magnetic field is negative (${{\rm{\varepsilon }}}_{//} < 0$). The density of the plasma layer facing the antenna is far from being spatially homogeneous and constant with time. There is usually a strong radial density gradient in the vicinity of the antenna with a typical gradient length of a few centimeters, shorter than the LHRF wavelength. However RF modeling shows that the coupling properties depend weakly on the gradient and, moreover, the simplification of the dielectric tensor (ε_⊥_ ≈ 1 and ε_A_ ≈ 0) still holds for the plasma layer close to the antenna, which matters for the coupling of the wave. In high performance plasma, instabilities occur in the plasma; the density at the plasma edge varies periodically with time from a low value (ε_//_ ~ −1) to a high value (ε_//_ ~ −10)^4^ and can depart from the optimal coupling conditions achieved for $-3 < {{\rm{\varepsilon }}}_{//} < -2$. If a medium mimicking a homogeneous plasma is well-suited to fully determining the properties of an RF antenna, a complete characterization would require several loads to cover the range of useful densities. We present here a metamaterial load that is close to optimal coupling conditions (ε_//_ = −3) for LHRF waves.

We are led to materials whose permittivity is diagonal, in the form ${\boldsymbol{\varepsilon }}={{\rm{\varepsilon }}}_{0}\,\text{diag}\,(1,1,{{\rm{\varepsilon }}}_{//})$, where ${{\rm{\varepsilon }}}_{//} < 0$. These materials are called hyperbolic materials, since waves polarized in the *xy*-plane (TE polarization) behave as in a vacuum, whereas TM waves obey a hyperbolic dispersion relation (their dispersion curve is a hyperboloid)^5,6^. In our case the field entering into the plasma from the antenna propagates in the *xz*-plane. See supplementary information for more details.

One way to obtain a material with a diagonal permittivity ${\rm{\varepsilon }}={{\rm{\varepsilon }}}_{0}\,\text{diag}(1,1,{{\rm{\varepsilon }}}_{//})$ is to use an array of thin metallic wires that are parallel to the *z*-direction. Previous works^7,8^ have shown that in such metamaterials ${{\rm{\varepsilon }}}_{//}$ expresses using the Drude formula ε_//_ = 1 − ω_p_^2^/ω_0_^2^ with a plasma frequency ω_p_ depending on the wire’s dimensions and spacing, at least when the wavelength is much greater than the wire’s spacing, when the wavevector has no component along the *z*-direction (i.e. *k*_z_ = 0), and when the electric field is parallel to the *z*-direction. It is shown in ref.^9^ that these wire metamaterials have a strong spatial dispersion, i.e. ω_p_ (and consequently ε_//_ and the response of the medium) depends on the wavevector component along the wire direction.

Considering our case, it is clear from Fig. 1 that the antenna must emit waves with *k*_z_ ≠ 0. In our configuration, we use ${k}_{z}={k}_{//}\approx 2{k}_{0}=2{\omega }_{0}/c$, which is a convenient value for current drive applications in a tokamak. To this end, we use a phased array of waveguides antennas. It means that the previous design with parallel wires cannot be used. Then, in order to mimic the plasma we need to reduce the spatial dispersion of the metamaterial. Several solutions have been proposed for this^10^. We use the method of connecting the conducting wires of our metamaterial, which has been shown to limit the spatial dispersion^9^.

We designed and tested a fishnet load composed of stacked thin metallic grid layers. The fishnet structure has the important practical advantage that it can be built layer by layer. This kind of construction was previously used by some of the authors for very low ε_//_ medium to propagate a very low divergence beam in a vacuum^11,12^, and more recently to control the density of states in hyperbolic metamaterials^13^.

The fishnet metamaterial load is depicted in Fig. 1. It is made of a stack of thin copper grids. Each grid is parallel to the *yz*-plane with the following dimensions: 0.2 mm width of the conductors along *y* or *z*, 18 μm thickness along *x*, periods ${p}_{y}$ and ${p}_{z}$. The grids are printed on a 50 μm-thick polyimide film. They are spaced using Rohacell foam with low density (permittivity ${\rm{\varepsilon }}=1.04$) that gives a period ${p}_{x}$ along the *x*-axis.

The electromagnetic properties of the structure strongly depend on these dimensions. In order to obtain a structure with properties as close as possible to the expected hyperbolic material, we adjusted these parameters and tried to get dispersion curves as close as possible to those of a hyperbolic material with ${{\rm{\varepsilon }}}_{//}=-\,3$. Due to the symmetry of the excitation versus *y*, the set of phased waveguides generates waves in the plasma that have a ${k}_{y}$ component centered in ${k}_{y}=0$; the ${k}_{y}$ bandwidth can be reduced by increasing the dimension of the antenna along *y*, or by piling up several rows of waveguides. For this reason, we assume in the following that the spectrum of waves launched into the plasma has a negligible ${k}_{y}$ component. Since the excitation is at a given frequency, all the electromagnetic characteristics of the material related to wave propagation are contained in the equi-frequency dispersion curve for the components ${k}_{x}$ and ${k}_{z}$ of the wavevector.

**Figure 2:**
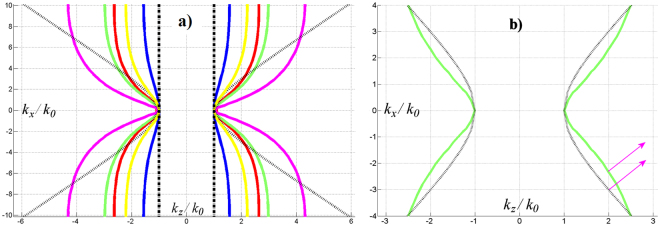
Equi-frequency dispersion curves at 3.7 GHz. (**a)** The gray hyperbola is the dispersion curve of a hyperbolic material with ${{\rm{\varepsilon }}}_{\perp }=1$ and ${{\rm{\varepsilon }}}_{//}=-3$. The colored curves are the dispersion curves of the grid-made metamaterial with periods ${p}_{x}=4\,\text{mm}$, ${p}_{z}=5.1\,\text{mm}$, and ${p}_{y}$ varying from 10 mm, 20 mm, 25 mm, 28 mm, 35 mm for blue, yellow, red, green and magenta curves respectively. The dashed black vertical lines correspond to the wired media. (**b)** Enlargement of the curve for ${p}_{y}=28\,\text{mm}$. The arrows show the average direction of energy propagation for ${k}_{z}=2{k}_{0}$.

Figure 2a shows the equi-frequency dispersion curves $({k}_{x},{k}_{z})$ for various dimensions of the fishnet cell. These curves are obtained by the eigenmode solver of CST Microwave Studio. To get these curves, we enforced the electric field to have no component along the *y*-axis. This is consistent with the way the material is excited (TE_10_ mode inside the waveguides). The gray hyperbola is the dispersion curve of a homogeneous plasma with ${{\rm{\varepsilon }}}_{\perp }={{\rm{\varepsilon }}}_{xx}={{\rm{\varepsilon }}}_{yy}=1$ and ${{\rm{\varepsilon }}}_{//}={{\rm{\varepsilon }}}_{zz}=-\,3$. Note that its equation is ${{\rm{\varepsilon }}}_{xx}\,{{k}_{x}}^{2}+{{\rm{\varepsilon }}}_{zz}\,{{k}_{z}}^{2}={{\rm{\varepsilon }}}_{xx}\,{{\rm{\varepsilon }}}_{zz}\,{{k}_{0}}^{2}$, i.e., ${{k}_{x}}^{2}-3\,{{k}_{z}}^{2}=-\,3\,{{k}_{0}}^{2}$. The colored curves show the dispersion curves of the fishnet metamaterials for several values of the period ${p}_{y}$. The two black vertical dashed lines are obtained when the wires along the *y*-direction are suppressed (the only wires are along the *z*-direction). As mentioned before, even if the effective parameters for a null *z*-component of the wavevector are correct, wire media fail to reproduce the dispersion relation of the homogeneous plasma for larger ${k}_{z}$ values. For our application, we are interested in propagative waves with *k*_z_ > *k*_0_, but no propagative waves exist inside the wire media for these wavevector values.

As shown in Fig. 2, for a fishnet metamaterial, the dispersion relation is not a perfect hyperbola. Thus, we had to make some choices: either choose parameters that give a curve as close as possible to the desired hyperbola in the range of ${k}_{z}$, or choose parameters that fulfill some other physical properties. We chose to build a metamaterial that will give a direction of energy propagation close to that of a homogeneous plasma. Note that the direction of energy propagation is normal to the dispersion curve^14^. Since the phased waveguide antenna launches waves with ${n}_{//}={k}_{z}/{k}_{0}\approx 2$, we retained the parameters associated with the green dispersion curve (Fig. 2b). This is a good compromise between a curve close to the hyperbola and one that gives for ${k}_{z}/{k}_{0}\approx 2$ the same energy propagation direction as the homogeneous plasma (magenta arrows).

The built structure is shown in Fig. 1. The dimensions of the grid layers are 396 mm (along the *z*-direction) × 168 mm (along the *y*-direction). To reduce the parasitic reflections of the electromagnetic field at the boundaries of the fishnet load, we surrounded the load by a 30 mm thick RF absorber foam on five of its faces, the sixth being the one where the waveguides feed the load.

**Figure 3:**
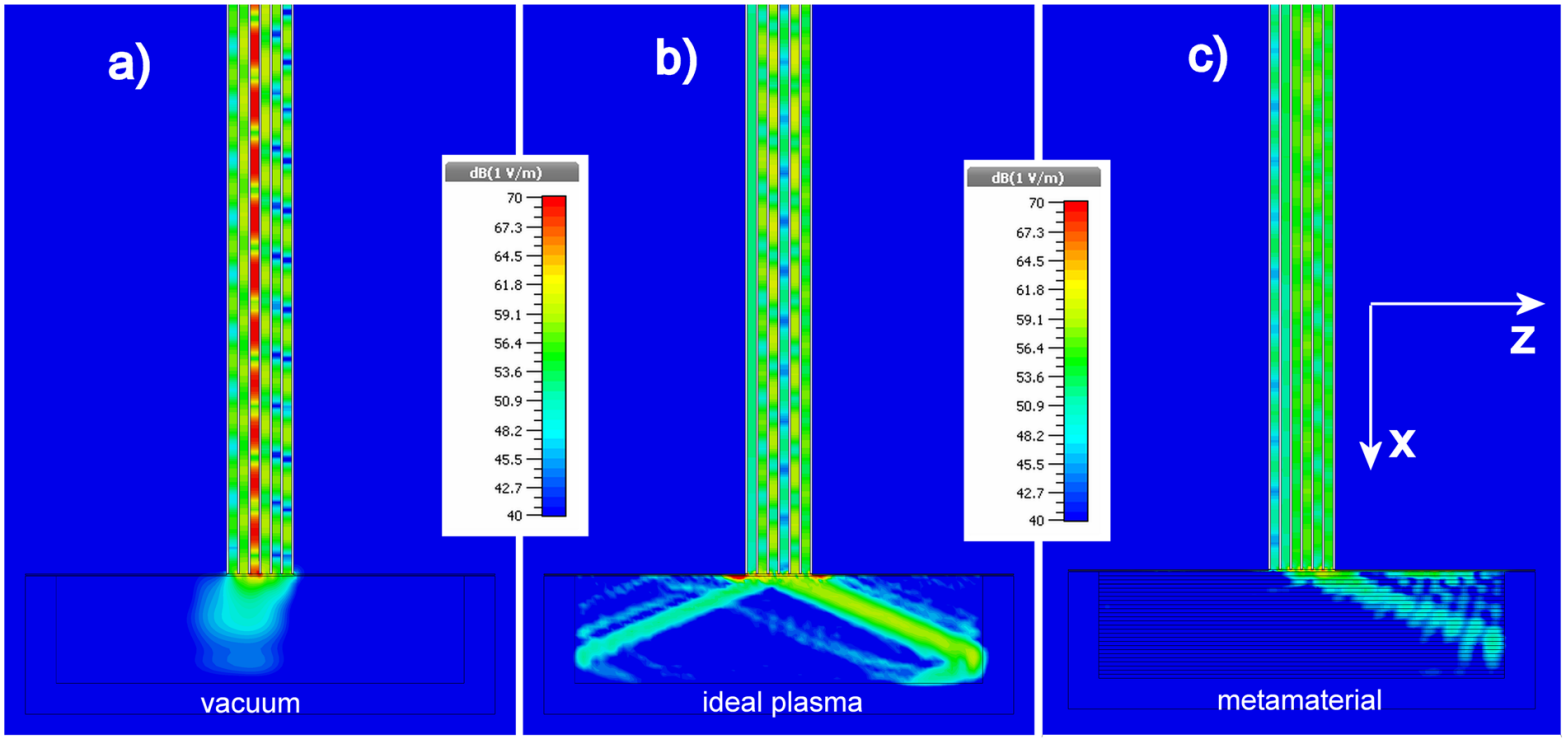
Numerical modeling of the modulus of the electric field at 3.7 GHz. The multi-junction antenna mouth is surrounded by a ground plane mimicking the tokamak vessel wall (horizontal line on these graphs). The field maps are shown in the middle of the antenna (y = 0 according to Fig. 1). The multi-junction antenna is loaded with vacuum (**a**), an ideal plasma with ${{\rm{\varepsilon }}}_{\perp }=1$ and ${{\rm{\varepsilon }}}_{//}=-\,3$. (**b**), and the fishnet metamaterial (**c**). A high resolution version of this graph showing the entire multi-junction antenna is given in supplementary information.

The antenna exciting the LHRF wave is composed of one input waveguide feeding 6 narrow phased waveguides stacked along the *z*-direction (see Fig. 3). This type of antenna, called a multi-junction antenna, allows reduction of the reflection coefficient in the input principal waveguide at the expense of an increase of the electric field in the narrow waveguides compared to a conventional antenna. The field emitted by this antenna has a spectrum centered on ${n}_{//}={k}_{z}/{k}_{0}\approx 2$. Consequently, there is no propagation inside the vacuum, a high reflection coefficient, and a high standing-wave ratio (SWR) inside the antenna, as shown in Fig. 3 left. Propagation inside the plasma is allowed according to Fig. 2(b). In this case, the SWR inside the antenna is low (Fig. 3, middle). The modeling depicted in Fig. 3 (right) shows that the fishnet metamaterial load that we designed preserves these properties: similar emitted field and low SWR. Moreover, the antenna emitted spectrum contains a secondary lobe centered on ${n}_{//}\approx -\,6$ (more details are given in supplementary). It can be seen in Fig. 2 that these waves can propagate in the ideal plasma (propagation direction on the left in Fig. 3b). In the case of the metamaterial, we can see from Fig. 2 that for *n*_//_ = −6 there is no existing propagative mode, as confirmed in Fig. 3c.

**Figure 4:**
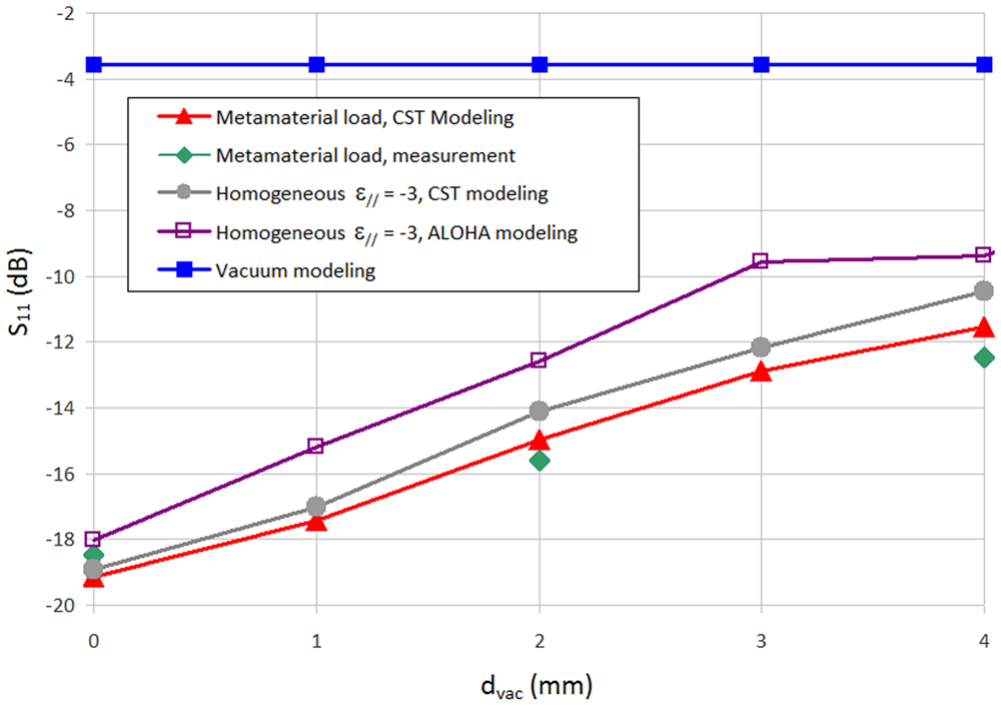
Reflection coefficient as a function of the gap between the antenna and the metamaterial load d_vac_. We also give the value of S_11_ when the antenna radiates in a vacuum. In the figure caption, “ε_//_ = −3” stands for the homogeneous load with ε_//_ = −3 and ε_⊥_ = 1.

To quantify the ability of our structure to fit to the plasma behavior, we measured the reflection coefficient *S*_11_ of the multi-junction antenna, and compared it (Fig. 4) to simulations using both CST Microwave Studio and the open-source code ALOHA^15^. CST is a full-wave frequency solver while ALOHA is a mode-matching code where the electromagnetic waves are fully absorbed in idealized semi-infinite plasma, which explains the small discrepancies between their results. We call *d*_vac_ the gap distance between the antenna and the load. As expected *S*_11_ is low when the metamaterial is close to the antenna and increases with *d*_vac._ Considering that in this case the results given by ALOHA have an uncertainty of approximately ±2 dB, one can note that there is a good agreement between the measurements and the modeling.

**Figure 5:**
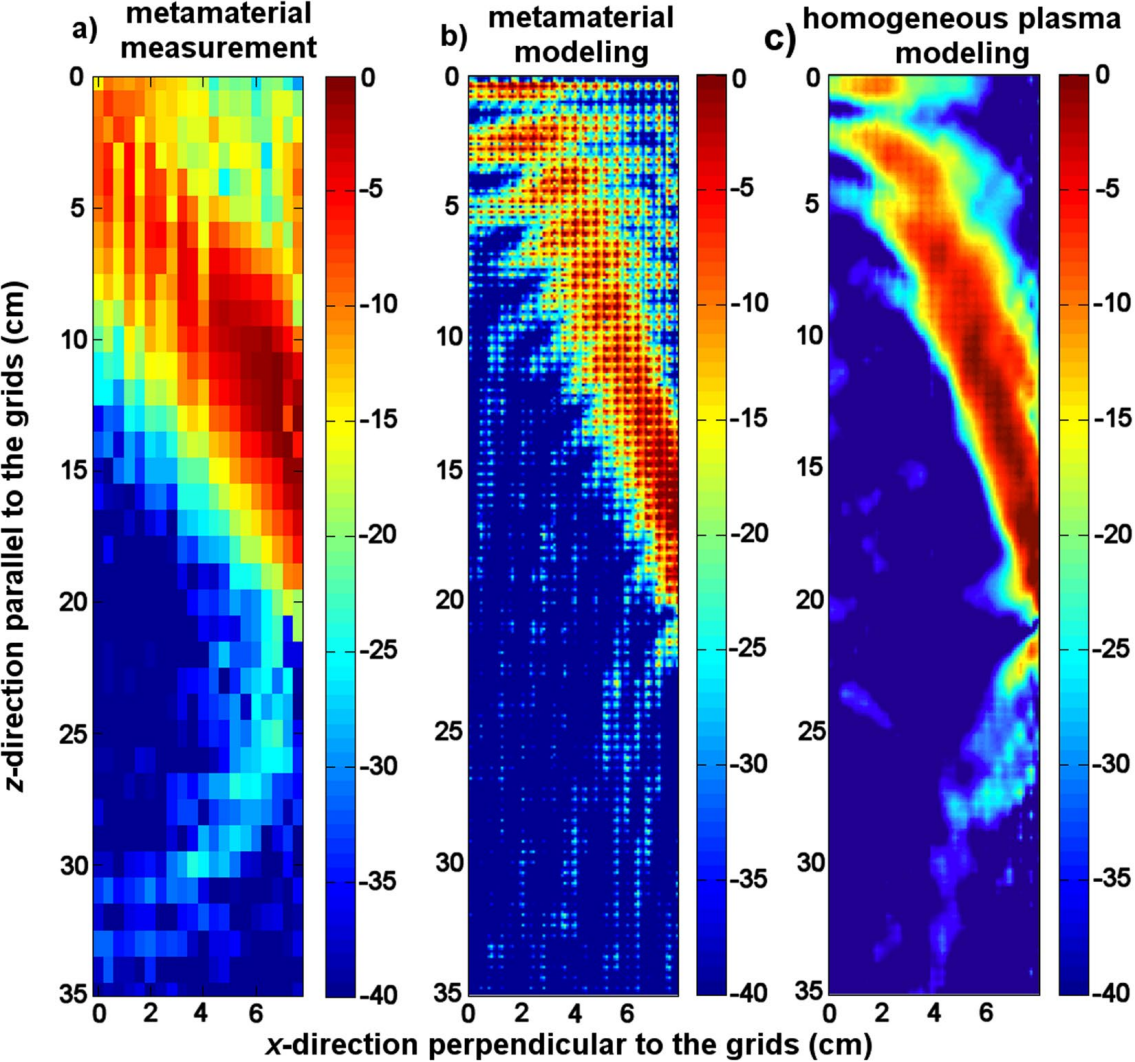
Magnetic field map with the metamaterial load (**a**) experiment, (**b**) modeling. The modeling of the equivalent plasma (ε_//_ = −3) is shown in (**c**).

The map of the x-component of the magnetic field propagating from the antenna to the medium was constructed from the measurements and compared to the simulations (Fig. 5). The main lobe of the launched spectrum (*n*_//_ = 2) is propagating along an angle α with the parallel direction (*z*-axis). The angle is given by ${\rm{\alpha }}={\tan }^{-1}{(\frac{{{\rm{\varepsilon }}}_{xx}({{\rm{\varepsilon }}}_{xx}-{n}_{z}^{2})}{{{\rm{\varepsilon }}}_{zz}{n}_{z}^{2}})}^{1/2}\approx 27\,\deg$, whereas the measurement gives α = 29 deg. The radiation pattern of the antenna obtained by Fourier transform of the field at its mouth shows secondary *n*_//_ lobes. The details are given in supplementary information. The highest amplitude secondary mode is for *n*_//_ = −6. This mode can be seen on Fig. 5c, and it propagates towards increasing *z*.

## Discussion and Conclusion

We numerically and experimentally show that the metamaterial load we designed matches well the properties of a homogeneous plasma with equivalent hyperbolic dielectric tensor, resulting in low reflection or low insertion losses for a typical LHRF antenna with *n*_//_ = 2. Experimental measurements and numerical modeling of magnetic field maps show that waves propagate in the anisotropic medium (ε_//_ = −3, ε_⊥_ = 1) along the expected directions. Thus, the metamaterial load will provide a drastic simplification for development of tokamak antennas and will help to make tokamak technology more efficient.

We envision many different versions and improvements. In a next step, two loads at 5 GHz with ε_//_ = −10 and −1 will be manufactured using the same scheme. These loads will be tested with a simple 4-waveguide antenna.

New concepts of antenna will also be tested with these loads. From a longer term perspective, several refinements can be proposed. For instance, a load mimicking a typical density gradient along the *x*-axis (radial) of a tokamak plasma near the antenna (Δε_//_/Δ*x* ~ −10/5 cm) can be obtained by continuously varying the dimensions of the metallic grid across the 25 layers. Gyrotropic properties that we neglected, but could be important for some frequency ranges could also be simulated by specific metamaterials structures^16,17^. Moreover, active metamaterials could reproduce the dynamics and instabilities of the plasma^18^. Finally, such anisotropic structures can be used in optical domain to enhance spontaneous emission, negative refraction and superlensing effects^5^.

## Methods

The load is tested with a 6-waveguide multi-junction-type antenna module. The individual waveguides (dimensions 8 mm × 72 mm) are phased with built-in phase shifters (ΔΦ = π/2) in order to launch a wave with a parallel wave index centered on *n*_//_ = 2.0. The height of the waveguides is such that all modes, except TE_10_, are evanescent. Due to the limited number of waveguides the *n*_//_ spectrum is rather broad (Δ*n*_//_)_mid-height_ = λ_0_/*L*_*//*, ant_ ≈ 1. A detailed analysis of the emitted spectrum is presented in supplementary information and shows that in addition a secondary lobe exists for *n*_//_ = −6. The distance between the antenna and the load, which is terminated by one half of a period ${p}_{x}/2$ of foam layer, needs to be accurately tuned as the wave (*n*_*//*_ > 1) is evanescent in vacuum/air with an evanescent length in the mm range. However, because of the rather poor flatness and thickness accuracy of the layers of spacers and absorbers, the mean gap cannot be controlled with accuracy better than 0.5–1 mm.

The RF field in the load was measured in a plane at mid-height of the waveguides (*y* = 0) where the amplitude of the TE_10_ mode is the highest. The field was measured with a magnetic loop (ϕ = 3 mm) inserted inside the load through empty channels hollowed in the foam.

The experimental results are compared to those obtained by full wave modeling, using either an open-source code ALOHA including a plasma module^15^ or the commercial RF code CST Microwave Studio, which can also describe the wave propagation in homogeneous plasma.

## Electronic supplementary material

Supplementary information
