# Supplementary material for: Mimicking Electromagnetic Wave Coupling in Tokamak Plasma with Fishnet Metamaterials
Source: Sci Rep. 2018 Apr 11;8:5841. doi: 10.1038/s41598-018-24250-0 (PMC5895602; doi:10.1038/s41598-018-24250-0)
Supplement: Supplementary file 1 — Supplementary information [file 41598_2018_24250_MOESM1_ESM.pdf]

MIMICKING ELECTROMAGNETIC WAVE COUPLING IN TOKAMAK PLASMA WITH FISHNET METAMATERIALS

K. Rustomji, R. Abdeddaim, J. Achard, M. Chmiaa, E. Georget, M. Goniche, W. Helou, J. Hillairet, S. Enoch, G. Tayeb

SUPPLEMENTARY INFORMATION

Propagation inside the plasma

The axes *x,y,z* are those used in the previous part of the paper (see Fig. 1) and $(\mathbf{e}_x, \mathbf{e}_y, \mathbf{e}_z)$ are the unit vectors along these axes.

We consider, inside a medium with permittivity

$$[\varepsilon] = \begin{bmatrix} \varepsilon_{xx} & 0 & 0 \\ 0 & \varepsilon_{yy} & 0 \\ 0 & 0 & \varepsilon_{zz} \end{bmatrix}$$

the propagation of a plane wave with electric field (time dependence in $\exp(-i\omega t)$)

$$\mathbf{E}(x,z) = \mathbf{E}_0 \exp\left(i\,\mathbf{k}\bullet\mathbf{r}\right)$$

where $\mathbf{k} = k_0(n_x\mathbf{e}_x + n_z\mathbf{e}_z)$.

The wave equation

$$-\mathbf{k}\times\left(\mathbf{k}\times\mathbf{E}\right) = k_0^2\,[\varepsilon]\mathbf{E}$$

leads to

$$\begin{bmatrix} \varepsilon_{xx} - n_z^2 & 0 & n_x n_z \\ 0 & \varepsilon_{yy} - n_x^2 - n_z^2 & 0 \\ n_x n_z & 0 & \varepsilon_{zz} - n_x^2 \end{bmatrix} \begin{bmatrix} E_x \\ E_y \\ E_z \end{bmatrix} = 0$$

This equation splits into two separate equations:

$$\left(\varepsilon_{yy} - n_x^2 - n_z^2\right)E_y = 0 \qquad (1)$$

and

$$\begin{bmatrix} \varepsilon_{xx} - n_z^2 & n_x n_z \\ n_x n_z & \varepsilon_{zz} - n_x^2 \end{bmatrix} \begin{bmatrix} E_x \\ E_z \end{bmatrix} = 0 \qquad (2)$$

The solutions of the first equation (1) are plane waves with electric field parallel to $\vec{e}_y$ and their dispersion relation is that of a homogeneous media with permittivity ε_{yy} :

$$n_x^2 + n_z^2 = \varepsilon_{yy}$$

In our present problem, we can consider that these waves are not present, because the antenna generates almost no wave with this polarization.

The second equation (2) admits solutions $\mathbf{E} = \left(E_x, 0, E_z\right)$ polarized in the plane $(\vec{e}_x, \vec{e}_z)$, provided that its determinant vanishes, which gives their dispersion equation:

$$\varepsilon_{xx} n_x^2 + \varepsilon_{zz} n_z^2 = \varepsilon_{xx}\varepsilon_{zz}$$

Spectrum generated by phased waveguides

In this appendix, we give a rough description of the spectrum of the waves launched into the plasma by a set of phased waveguides. The waveguides are in the half space $x<0$, their terminations are in the plane $x=0$, and we assume that anywhere else in this plane $x=0$ there is a perfect conducting plate. The field emitted by these waveguides is launched in the half-space $x>0$. We make some simplifying hypotheses; we assume that the waveguides apertures are infinitely extended along the *y*-axis. This means that the problem becomes *y*-independent, and the field emitted can be written as a plane wave packet

$$\mathbf{E}(x,z)=\int_{-\infty}^{+\infty}\mathbf{A}(k_z)\exp\left(i(k_x x+k_z z)\right)dk_z$$

We denote by *b* the waveguide aperture along the *z*-axis. We denote by Δz the distance between the centers of two adjacent waveguides along the *z*-axis. We denote by $\Delta\phi$ the phase shift between the fields emitted by two adjacent waveguides. We denote by *N* the numbers of waveguides (*N* = 6 and $\Delta\phi=\pi/2$ in our practical case). We assume that the electric field in the waveguide termination has constant amplitude and a linear polarization along *z*.

If we denote by $\Pi(u)$ the rectangular function with width *b*, equal to 1 for $-b/2<u<b/2$ and vanishing anywhere else, the electric field in the plane $x=0$ where the waveguides end is the function

$$E_z(x=0,z)=\sum_{n=1}^{N}\exp(in\,\Delta\phi)\,\Pi(z-n\,\Delta z)$$

The spectrum $A_z(k_z)$ emitted by the set of waveguides in the half-space $x>0$ is such that

$$E_z(0,z)=\int_{-\infty}^{+\infty}A(k_z)\exp\left(ik_z z\right)dk_z$$

and consequently

$$A(k_z)=\frac{1}{2\pi}\int_{-\infty}^{+\infty}E_z(0,z)\exp\left(-ik_z z\right)dz$$

After some calculations, and putting

$$k_z=k_0\,n_z=\frac{2\pi}{\lambda_0}n_z$$

$$X=\exp\left(i(\Delta\phi-k_0\,\Delta z\,n_z)\right)$$

we get

$$A(n_z)=\frac{X^{N+1}-X}{X-1}\;\frac{b}{2\pi}\;\frac{\sin\left(\frac{k_0\,b\,n_z}{2}\right)}{\frac{k_0\,b\,n_z}{2}}$$

Finally, if we put

$$\mathrm{sinc}(x)=\frac{\sin(\pi x)}{\pi x}$$

the spectral density of the waves launched by the set of waveguides is

$$\left|A(n_z)\right|^2=\left|\frac{X^{N+1}-X}{X-1}\right|^2\left(\frac{b}{2\pi}\right)^2\ \mathrm{sinc}^2\left(\frac{b\,n_z}{\lambda_0}\right)$$

The Supplementary Fig.1 gives the spectral density at 3.7 GHz in two cases:

a) $N=6$ waveguides with width $b=8\text{ mm}$, spacing $\Delta z=10\text{ mm}$ and phase shift $\Delta\phi=\pi/2$

b) $N=1$ waveguide with width $b=8\text{ mm}$

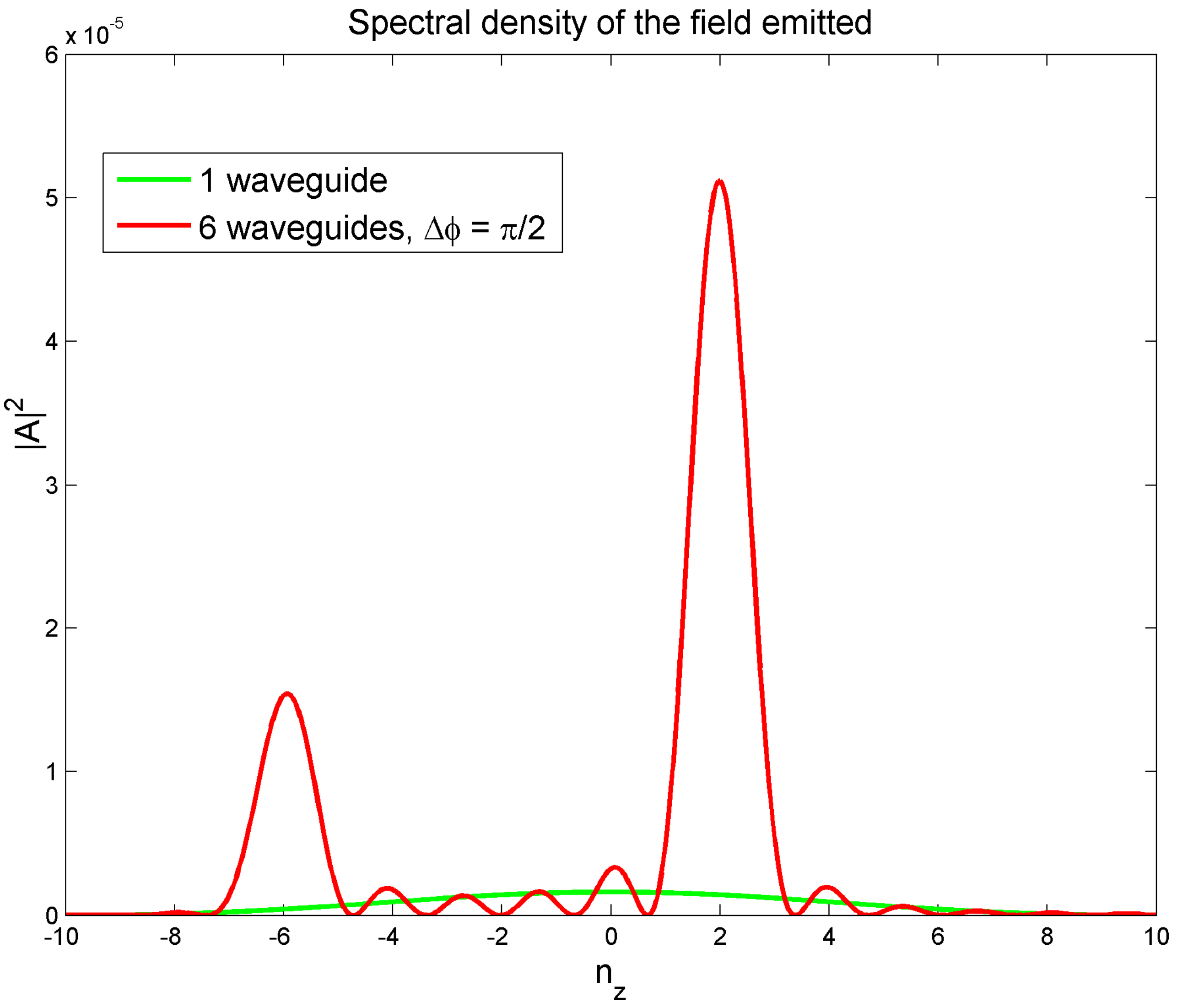

Supplementary Fig.1. Spectral density of the field emitted by phased waveguides.

The position of the peaks can be easily retrieved using well known diffraction gratings properties. When N tends to infinity, the set of phased waveguide apertures give birth to grating orders and the separation between the orders is $\Delta k_z = 2\pi / \Delta z$, i.e., $\Delta n_z = 2\pi / (k_0 \; \Delta z)$. The position of the first order ($m=0$) is linked with the phase shift $\Delta\phi$: $n_z(0) = \Delta\phi / (k_0 \; \Delta_z)$. Consequently, the positions of the peaks are given by

$$n_z(m) = n_z(0) + m \frac{2\pi}{k_0 \; \Delta z} = \frac{\Delta\phi}{k_0 \; \Delta z} + m \frac{2\pi}{k_0 \; \Delta z}$$

In our case, at a frequency of 3.7 GHz, $\Delta z = 10^{-2}$ m, and $\Delta\phi = \pi / 2$, this formula gives the position of the two major peaks: $n_z(0) = 2.03$ and $n_z(-1) = -6.08$.

Picture of a real LHRF antenna

The picture in Supplementary Fig. 2 shows a LHRF antenna in the vessel of the Tore Supra tokamak. The dimensions of the array of 6 × 48 waveguides are 580 mm ×580 mm.

The multi-junction antenna that has been used for our measurements simulates one module of 6 waveguides. Both antenna provide an emitted spectrum centered on $k_z = 2k_0$.

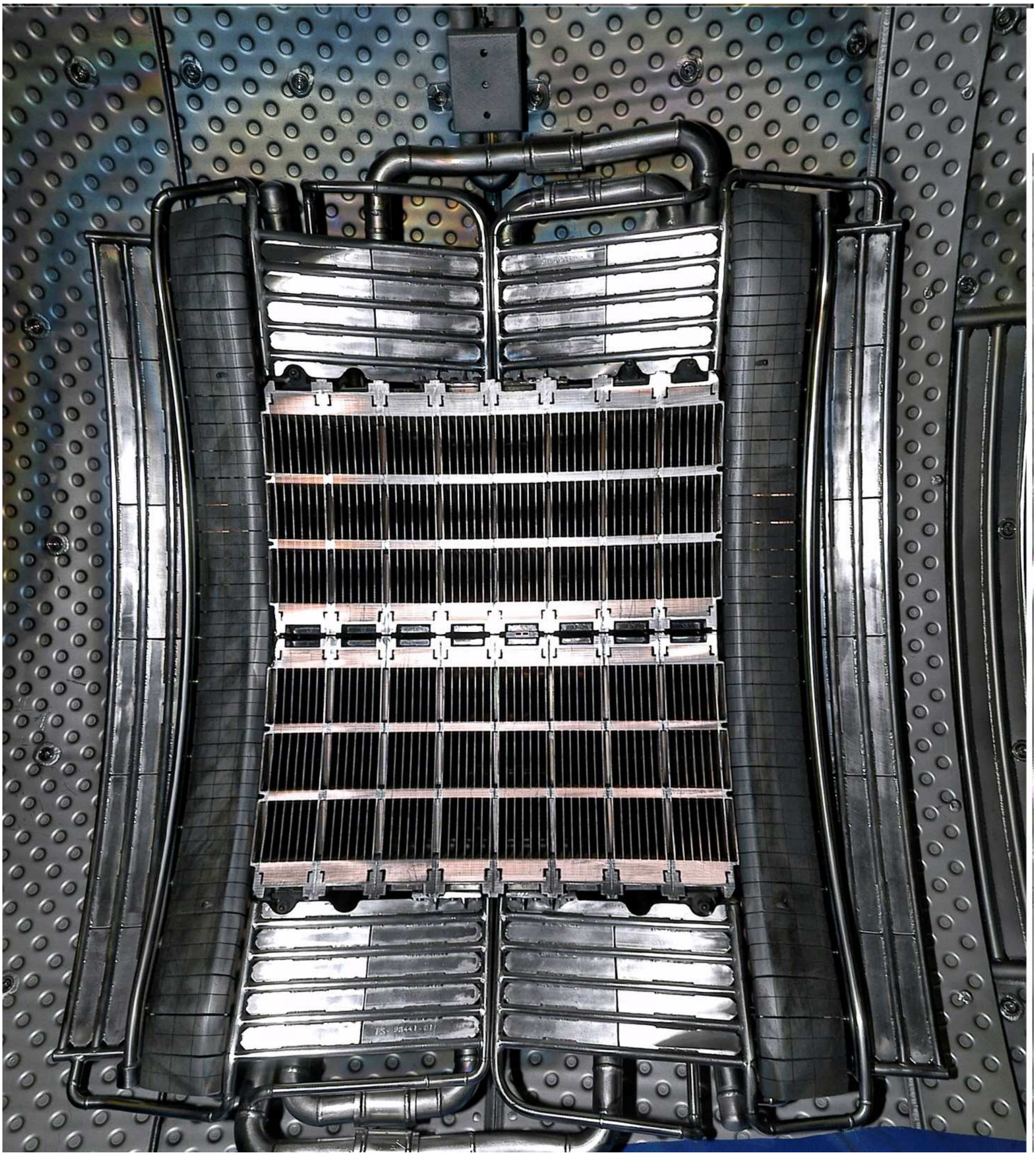

Supplementary Fig.2. Picture of a LHRF antenna.

Numerical modeling of the entire multi-junction antenna

Supplementary Fig. 3 is the complete version of Fig. 3. It shows the entire multi-junction antenna. This antenna is fed from the top. The first section splits into three waveguides, then into six waveguides, with the required phase shifts between adjacent waveguides. The same color scale is used for the three graphs. The vacuum load generates high reflection coefficients and a high standing wave ratio inside the waveguides. The "ideal plasma" and the metamaterial load generate much less standing wave ratio.

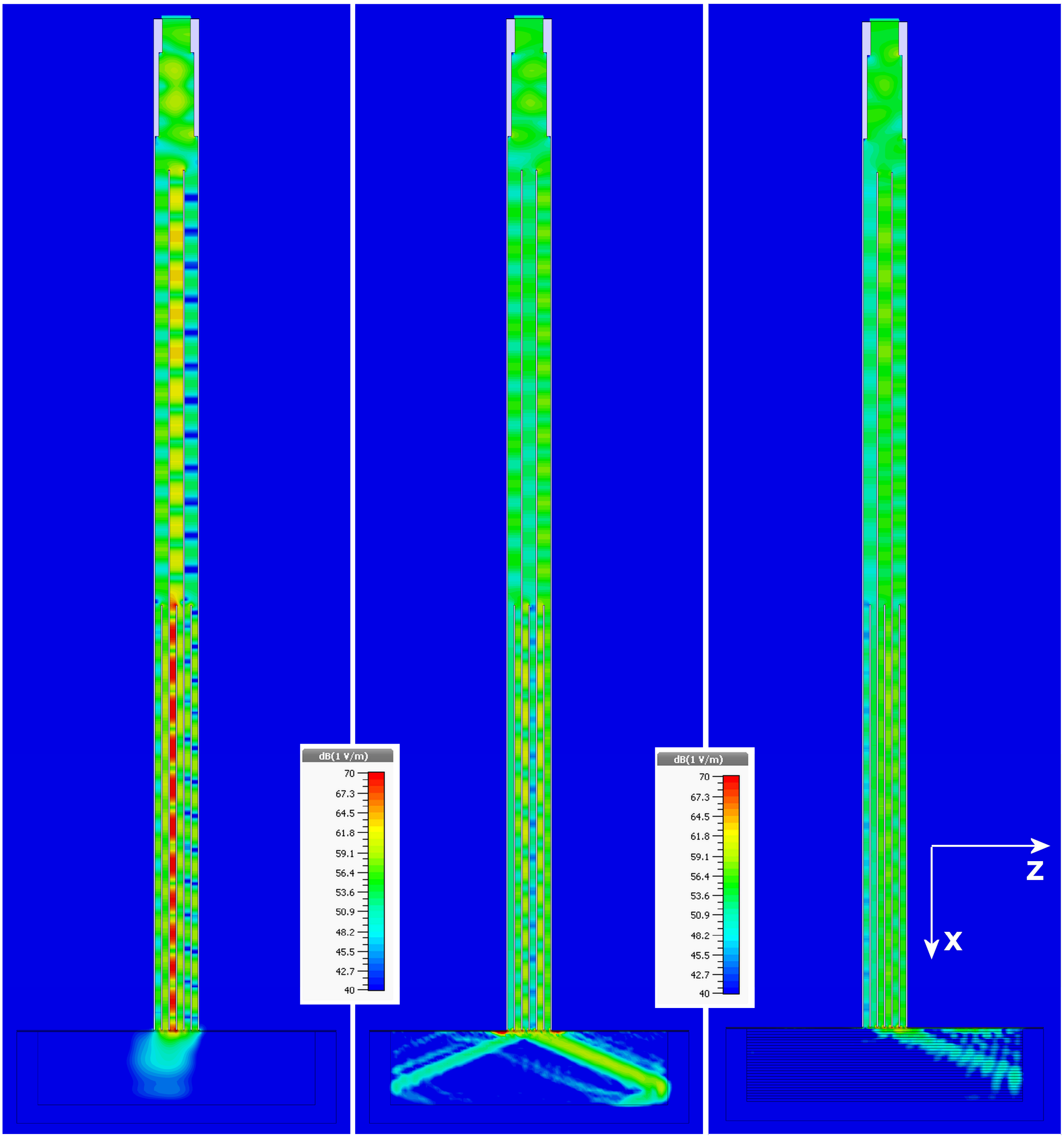

Supplementary Fig.3. Numerical modeling of the modulus of the electric field at 3.7 GHz. The multi-junction antenna mouth is surrounded by a ground plane (horizontal line on these graphs). The field map is in the middle of the antenna (y = 0 according to Fig. 1). The multi-junction antenna is loaded with vacuum (left), an ideal plasma with $\varepsilon_{\perp} = 1$ *and* $\varepsilon_{//} = -3$ *(middle), and the fishnet metamaterial (right).*
